# Supplementary material for: MetaRibo-Seq measures translation in microbiomes
Source: Nat Commun. 2020 Jun 29;11:3268. doi: 10.1038/s41467-020-17081-z (PMC7324362; doi:10.1038/s41467-020-17081-z)
Supplement: Supplementary file 10 — Supplementary Data 7 [file 41467_2020_17081_MOESM10_ESM.zip › File2/Confidence_High_Taxonomy/309552_out.krona.html]

Javascript must be enabled to view this page.

members
magnitude
magnitudeUnassigned
count
unassigned
taxon
rank

309552\_out

2

superkingdom
2
2

1239
phylum
2

class
1
186801

1
order
186802

186803
1
family

1
genus
1432051

1
species

SRS146888\_contig\_number\_5574
1720294

526524
class
1

order
1
526525

128827
1
family


SRS017521\_contig\_number\_3594
species
1
469614
